# The impact and importance of achieving a complete haematological response prior to renal transplantation in AL amyloidosis

**DOI:** 10.1038/s41408-020-0325-2

**Published:** 2020-05-22

**Authors:** Oliver C. Cohen, Steven Law, Helen J. Lachmann, Faye Sharpley, Sriram Ravichandran, Shameem Mahmood, Sajitha Sachchithanantham, Carol J. Whelan, Ana Martinez De Azcona Naharro, Marianna Fontana, Philip N. Hawkins, Julian D. Gillmore, Ashutosh D. Wechalekar

**Affiliations:** National Amyloidosis Centre, University College London, London, UK

**Keywords:** Haematological cancer, Disease-free survival, Therapeutics

Dear Editor,

Renal dysfunction is a common presenting feature of AL amyloidosis and one-third of affected patients will develop end-stage renal failure (ESRF)^1^. Consequently, a sizable proportion of patients will require either renal replacement therapy (RRT) or renal transplantation.

In AL amyloidosis, achieving a deep haematological response (HR) to therapy (at least a very good partial response (VGPR)^1^ or a 90% reduction in the difference between free light chains (dFLC)^2^) improves renal outcomes and survival. Renal transplantation is associated with lower mortality, less risk of cardiovascular events and improved quality of life when compared to RRT^3^. In 2010, we reported a median overall survival (OS) of 6.5 years and median graft survival of 5.8 years^4^ in patients with AL amyloidosis who underwent renal transplantation, whilst a recent report from the Boston group documented an impressive 10.5 year OS and 8.3-year graft survival^5^, perhaps reflecting advances in patient selection and available therapies over the last decade. Appropriate patient selection is imperative from the perspective of both fitness to undergo transplantation and depth of HR. Assessment of HR in patients with ESRF provides a challenge due to the polyclonal increase in serum-free light chains (sFLC) that occurs in renal failure^6^.

We report here fifty patients with AL amyloidosis who underwent renal transplantation, identified from the database of the UK National Amyloidosis Centre, over the last 15 years. Patients transplanted prior to this were excluded given the marked changes in treatments and poorer outcomes in earlier years. Five patients had renal transplants for reasons unrelated to amyloidosis, 4 patients were lost to follow up and 1 patient had a renal allograft abroad prior to their first presentation to the NAC. Forty patients were evaluable for outcomes. The diagnosis of AL amyloidosis was confirmed by central review of histological material inclusive of Congo red staining of the renal biopsy. The amyloid subtype was subsequently confirmed by immunohistochemistry with specific antibodies, or by mass spectrometry.

Prior to renal transplantation, haematological responses were defined as per the previous literature^7^. In the setting of ESRF, raised levels of free kappa and lambda light chains may reflect slowed renal light chain clearance and not the presence of a monoclonal gammopathy^8^ thus a seemingly abnormal ratio using the standard reference range (0.26–1.65 mg/L) may be seen in patients with a fully suppressed plasma cell clone. A wider “normal” range for sFLC has been suggested for use in renal failure (0.37–3.1 mg/L)^8,9^ to better account for this polyclonal sFLC increase. Responses were also assessed using this wider renal sFLC range to define complete response (CR). In the post-transplant setting, HR was assessed as per international consensus criteria^10^. Organ involvement was determined as per consensus guidelines^11^. Given that LV mass increases and is prognostic in uraemic cardiomyopathy^12^, LV wall thickness of <12 mm, ≥12 mm or ≥13 mm were assessed. Due to the selected patient population, the numbers of patients with an LV wall thickness above 14 mm were too small for meaningful analysis.

Progression-free survival (PFS) was defined as time from renal transplantation to haematological progression or death whilst OS was calculated from the date of renal transplantation to death from any cause. Renal graft survival was calculated as time from renal transplantation to recurrence of ESRF (death without graft failure was censored).

Patient characteristics are summarised in Supplementary Table 1. At presentation, 12 (30%) patients were on RRT. A further 24 (60%) progressed to ESRF and required RRT prior to renal transplantation whilst 4 (10%) were transplanted pre-emptively. Median time from diagnosis to ESRF was 15 months (0–115 months) and from RRT to renal allograft was 28 months (3–83 months). Twenty patients (50%) received cadaveric transplants whilst the remainder had a live donor.

Haematological responses at the time of renal transplantation and on re-assessment immediately post-transplant were: CR—24 (60.0%)/27 (67.5%), VGPR—6 (15.0%)/8 (20.0%), partial response (PR)—6 (15.0%)/2 (5.0%) and no response (NR)—3 (7.5%)/1 (2.5%), respectively. No patient received chemotherapy between the pre- and post-renal transplantation sFLC measurements. Prior to renal transplantation, one patient was excluded as their light chains were not evaluable whilst post-transplantation, one patient died after renal transplantation before these readings were taken. Based on post-renal transplant sFLC measurements, 7 patients (17.5%) had their HR re-classified, of which 6/7 (85.7%) were assigned an improved response category including two patients who had been classed as non-responders pre-transplantation. When the wider “renal” sFLC normal range^9^ was applied to pre-transplant response assessment, there was no change in the assigned depth of response following renal transplantation. Outcomes were studied using dFLC value (<10 mg/L), percentage reduction (>90%) and involved free light chain (iFLC) absolute reduction (<20 mg/L). There were no significant differences between responders and non-responders using pre-transplantation sFLC results. Using post-transplant clonal markers, OS was significantly better in patients with a deeper response assessed either as standard CR or dFLC <10 mg/L, iFLC <20 mg/L or dFLC reduction of >90% compared to standard VGPR or less or a lesser depth of d/iFLC response, respectively (Table 1).

**Table 1:** Assessment of haematological response pre- and post-renal transplantation and its impact on overall survival.

	Pre-Renal Transplantation		Post-Renal Transplantation	
	OS from renal transplant	*P* value	OS from renal transplant	*P* value
CR	123.9 (72.8–143.2) months	0.015	137.8 (113.3–161.6) months	0.0001
VGPR or worse	70.4 (38.4–117.6) months		58.9 (34.4–91.6) months	
dFLC >90% reduction	114.4 (47.6–140.4) months	0.602	133.5 (109.3–158.4) months	0.0001
dFLC <90% reduction^2^	92.9 (63.9–122.1) months		64.4 (41.9–114.1) months	
dFLC <10 mg/L	Nil Patients		137.8 (112.4–163.2) months	0.0001
dFLC >10 mg/L^14^			68.5 (58.7–97.3) months	
iFLC <20 mg/L	Nil Patients		150.3 (126.2–174.4) months	0.034
iFLC >20 mg/L^15^			84.3 (58.9–103.1) months	

*SFLC* serum free light chain, *OS* overall survival, *CR* complete response, *VGPR* very good partial response, *dFLC* difference in free light chains, *iFLC* involved free light chains.

The median follow up from renal transplantation was 8.9 years (2.2–19.3 years). During the period of follow up, 13 (32.5%) patients died. One patient died within a few days of renal transplantation due to a post-operative hypotensive event and was known to have Mayo Stage 3 cardiac amyloidosis. Of the remaining deaths, 5 patients died in relapse, 5 died whilst in haematological remission and 2 are unknown. From renal transplantation, haematological PFS was 6.9 years (95% CI 5.1–8.7 years) and median OS was 9.0 years (95% CI 5.5–10.1 years). Patients who achieved a CR, based on pre-transplant sFLCs, achieved a markedly higher PFS (8.5 years; 95% CI 5.7–11.4 years) (*p* = 0.024) and OS (10.3 years; 95% CI 6.1–11.9 years) (*p* = 0.015) (Fig. 1). In contrast, patients achieving a pre-transplant VGPR, did not have significantly different PFS (*p* = 0.293) or OS (*p* = 0.106) compared with those patients who were given a renal transplant in a lesser HR (PR/NR).

**Fig. 1:**
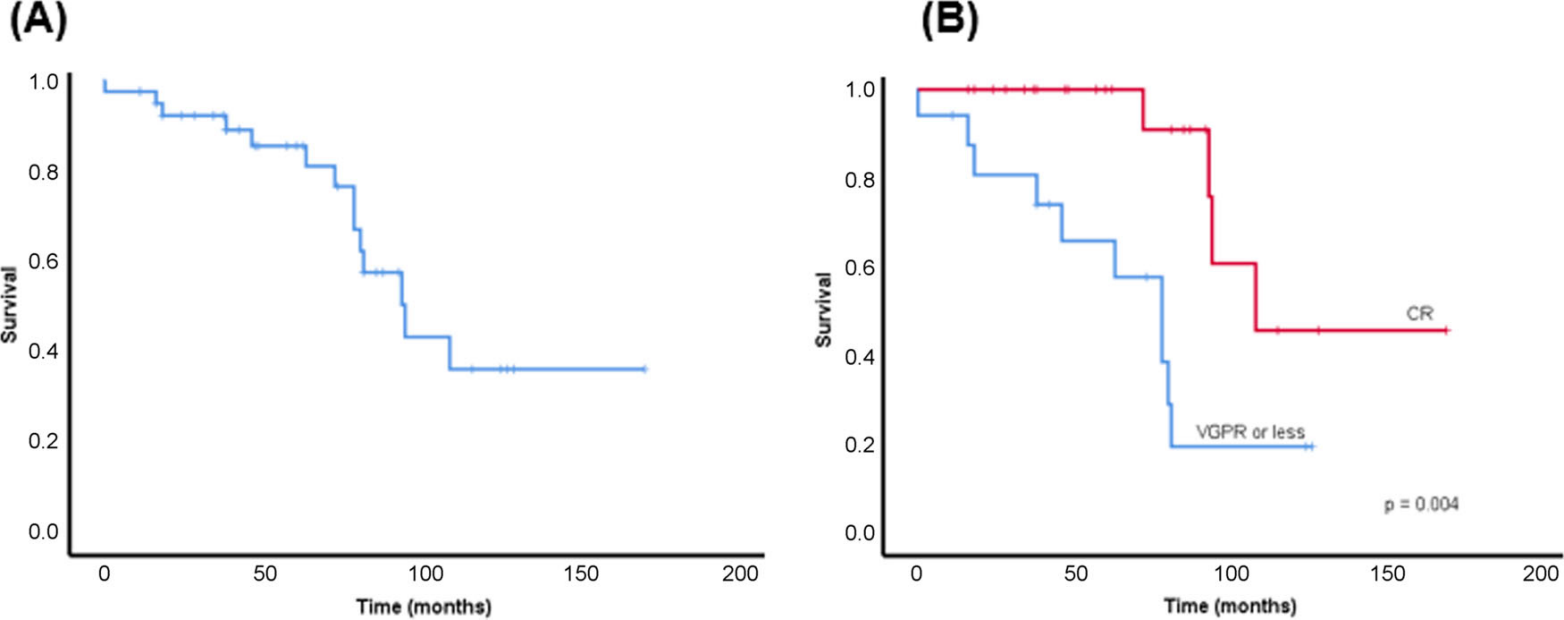
Survival from renal transplantation. **a** Overall survival **b** Overall survival by haematological response.

Prior autologous stem cell transplantation, number of prior lines of therapy and source of renal transplant (live vs. cadaveric) did not impact survival. Cardiac outcomes were assessed using the usual amyloid definition (mLV wall >12 mm) or a higher renal threshold (mLV wall 13 mm) given that increased LV wall thickness is both common and prognostic in end-stage renal failure^12^. All patients with thicker LV walls (>12 or >13 mm) had a higher hazard ratio for poorer outcomes but it was significantly worse for those with an LV wall >13 mm (median OS 9.7 years [7.8–11.7 years] vs. 3.6 years [0.7–6.6 years] for LV wall > or <13 mm respectively (*p* = 0.01; HR 9.60 [2.08–44.23]). This highlights the importance of patient selection and pre-transplant cardiac status. It is unclear whether this survival difference reflects a higher cardiac amyloid burden, uraemic cardiomyopathy or a combination. The only peri-operative death occurred in a patient with Mayo stage 3 cardiac amyloidosis who had achieved a good haematological and cardiac response. Following this patient, our centre routinely undertakes functional stress cardiac testing in all patients with abnormal baseline echocardiograms being considered for renal transplant.

On an intention-to-treat basis, median graft survival was 12.4 years (95% CI 10.7–14.2 years). There were 2 acute graft failures within 4 weeks of implantation. On long term follow up, only one patient lost their graft and required initiation of RRT. Three patients had proven amyloid recurrence in the renal graft on repeat biopsy without graft loss. A further 2 patients had evidence of amyloid recurrence on ^123^I-labelled serum amyloid P component (SAP) scintigraphy without recurrence of proteinuria or deterioration in renal function. Nine (22.5%) patients required further chemotherapy for AL amyloidosis due to post-transplant haematological relapse, or a post-transplant lymphoproliferative disorder in one case, of which one patient (11.1%) had evidence of increasing proteinuria. The remainder had evidence of haematological relapse only without organ progression. There was no difference in graft survival (*p* = 0.350) or OS (*p* = 0.788) in patients who received chemotherapy following graft implantation.

In summary, the literature on renal transplant outcomes in AL amyloidosis is relatively sparse. Whilst this study is limited by its retrospective nature and relatively small patient numbers, we demonstrate excellent long term patient survival with renal transplantation and that renal graft failure secondary to AL amyloidosis is uncommon. Patient survival is dictated by HR and LV wall thickness as opposed to graft failure; highlighting the importance of careful assessment of both (HR and LV wall thickness) when determining whether a patient is suitable for renal transplantation. Use of the renal FLC threshold to assess HR prior to transplantation shows promise but requires further validation. Whilst FLC levels are important, a more comprehensive clonal disease assessment including serum/urine electrophoresis and immunofixation as well as a bone marrow biopsy may be needed. Novel mass spectrometric methods of identifying the monoclonal component of the sFLC are likely to provide a solution but are not yet routinely available^13^. Renal transplantation should be considered more often in patients with AL amyloidosis predominant renal involvement in ESRF.

## Supplementary information

Patient Baseline Characteristics

## References

[1] Kastritis E (2017). Renal outcomes in patients with AL amyloidosis: Prognostic factors, renal response and the impact of therapy. Am. J. Hematol..

[2] Pinney JH (2011). Outcome in renal Al amyloidosis after chemotherapy. J. Clin. Oncol..

[3] Tonelli M (2011). Systematic review: kidney transplantation compared with dialysis in clinically relevant outcomes. Am. J. Transplant..

[4] Sattianayagam PT (2010). Solid organ transplantation in AL amyloidosis. Am. J. Transplant..

[5] Angel-Korman A (2019). Long-term outcome of kidney transplantation in AL amyloidosis. Kidney Int..

[6] Fraser SDS (2017). The association of serum free light chains with mortality and progression to end-stage renal disease in chronic kidney disease: Systematic review and individual patient data meta-analysis. Mayo Clin. Proc..

[7] Pinney JH (2013). Renal transplantation in systemic amyloidosis-importance of amyloid fibril type and precursor protein abundance. Am. J. Transplant..

[8] Abadie JM, van Hoeven KH, Wells JM (2009). Are renal reference intervals required when screening for plasma cell disorders with serum free light chains and serum protein electrophoresis?. Am. J. Clin. Pathol..

[9] Hutchison CA (2008). Quantitative assessment of serum and urinary polyclonal free light chains in patients with type II diabetes: an early marker of diabetic kidney disease?. Expert Opin. Ther. Targets..

[10] Palladini G (2012). New criteria for response to treatment in immunoglobulin light chain amyloidosis based on free light chain measurement and cardiac biomarkers: impact on survival outcomes. J. Clin. Oncol..

[11] Gertz MA (2005). Definition of organ involvement and treatment response in immunoglobulin light chain amyloidosis (AL): a consensus opinion from the 10th International Symposium on Amyloid and Amyloidosis, Tours, France. Am. J. Hematol..

[12] Foley RN (1995). The prognostic importance of left ventricular geometry in uremic cardiomyopathy. J. Am. Soc. Nephrol..

[13] Sharpley FA (2019). A novel mass spectrometry method to identify the serum monoclonal light chain component in systemic light chain amyloidosis. Blood Cancer J..

[14] Manwani Richa, Cohen Oliver, Sharpley Faye, Mahmood Shameem, Sachchithanantham Sajitha, Foard Darren, Lachmann Helen J., Quarta Cristina, Fontana Marianna, Gillmore Julian D., Whelan Carol, Hawkins Philip N., Wechalekar Ashutosh D. (2019). A prospective observational study of 915 patients with systemic AL amyloidosis treated with upfront bortezomib. Blood.

[15] Muchtar E (2019). Optimizing deep response assessment for AL amyloidosis using involved free light chain level at end of therapy: failure of the serum free light chain ratio. Leukemia..

