# Supplementary material for: The impact and importance of achieving a complete haematological response prior to renal transplantation in AL amyloidosis
Source: Blood Cancer J. 2020 May 22;10(5):60. doi: 10.1038/s41408-020-0325-2 (PMC7244514; doi:10.1038/s41408-020-0325-2)
Supplement: Supplementary file 1 — Patient Baseline Characteristics [file 41408_2020_325_MOESM1_ESM.docx]

**Table SI. Patient Baseline Characteristics**

| **Characteristic** | **N (%) (range)** |
| --- | --- |
| Age, median (range) | 53.5 (38-69) |
| Male, N (%)  Median ECOG | 24 (60)  1 (Range 0-3) |
| *Disease Isotype*  IgG  Light Chain Only  IgA  IgM | 20 (50)  16 (40)  2 (5)  2 (5) |
| Lambda restricted, N (%)  dFLC-R, median (range) | 28 (70)  112.7 (5.0-708.9) |
| Extra-renal Involvement  Cardiac  Liver  Peripheral Nerve  Autonomic Nerve  Gastrointestinal  Spleen*  Soft Tissue | 16 (40)  21 (52.5)  3 (7.5)  5 (12.5)  1 (2.5)  31 (77.5)  3 (7.5) |
| Baseline Organ Function  Creatinine  Median eGFR ml/min per 1.73m^2^, median (range)  Proteinuria, g per 24h, median (range)  NT-proBNP, ng/L, median (range)  ALP, IU/L, median (range)  Albumin, g/L, median (range) | 199μmol/L (69-756 μmol/L)  27ml/min  8.95g/24h (0.4-19.7g/24h)  1915.5ng/L (76-69999ng/L)  78 IU/L (19-1384 IU/L)  28g/L (12-46g/L) |
| CKD Stage at Diagnosis:  Stage I  Stage II  Stage III  Stage IV  Stage V | 2 (5)  8 (20)  8 (20)  10 (25)  12 (30) |
| Prior Lines of Therapy  Median (range)  ASCT | 2 (1-4)  10 (25) |

*Splenic involvement assessed by ^123^I-labelled serum amyloid P component (SAP) scintigraphy

Abbreviations: ECOG: Eastern Co-operative Oncology Group Performance Score, dFLC-R: ratio of difference between free light chains; eGFR: estimated glomerular filtration rate, NT-proBNP: N-terminal-proB-natruiretic peptide; ALP: alkaline phosphatase; CKD: chronic kidney disease; ASCT: autologous stem cell transplantation
